# Endothelial dysfunction and hemostatic imbalance in CAR T-cell-associated toxicities: pathophysiological insights and the role of circulating biomarkers

**DOI:** 10.3389/fimmu.2025.1699894

**Published:** 2025-11-03

**Authors:** Ana Belén Moreno-Castaño, Gontzal Iraola, Núria Martínez-Cibrián, Nil Albiol, Daniel-Nicolás Marco Prats, Julia Martinez-Sanchez, Pedro Castro, Maribel Diaz-Ricart

**Affiliations:** ^1^ Hemostasis and Erythropathology Laboratory, Hematopathology, Pathology Department, Centre Diagnòstic Biomèdic (CDB), Hospital Clínic Barcelona, Barcelona, Spain; ^2^ Institut d’Investigacions Biomèdiques August Pi i Sunyer (IDIBAPS), Barcelona, Spain; ^3^ Facultat de Medicina i Ciències de la Salut, Universitat de Barcelona, Barcelona, Spain; ^4^ Medical Intensive Care Unit, Hospital Clínic Barcelona, Barcelona, Spain; ^5^ Hematology Department, Institut del Càncer i Malalties de la Sang (ICAMS), IDIBAPS, Hospital Clínic Barcelona, Barcelona, Spain

**Keywords:** biomarkers, biomarker panels, CAR T toxicities, coagulopathy, CRS, ICANS, IEC-HS, prediction

## Abstract

Chimeric antigen receptor (CAR) T-cell therapy has revolutionized the treatment of relapsed or refractory hematologic malignancies. While its clinical efficacy is well established, CAR T-cell therapy is frequently associated with severe immune-mediated toxicities, including cytokine release syndrome (CRS), immune effector cell-associated neurotoxicity syndrome (ICANS), coagulopathy, and hemophagocytic lymphohistiocytosis-like syndrome (IEC-HS). Increasing evidence suggests that endothelial dysfunction, hemostatic imbalance, and complement activation are key contributors to the pathogenesis of these complications. Substantial research efforts have focused on identifying circulating biomarkers capable of predicting toxicity onset and severity, as well as stratifying patients at risk for early non-relapse mortality. In this review, we summarize the current understanding of the pathophysiological mechanisms underlying early CAR T cell–related toxicities, with particular emphasis on biomarkers of endotheliopathy and related pathways involved in their development. We focus on highlighting translational biomarkers with potential diagnostic, prognostic, and monitoring value that could be implemented in clinical practice to improve patient risk stratification, differential diagnosis, and therapeutic follow-up.

## 1 Introduction

Chimeric antigen receptor (CAR) T-cell therapy now constitutes a mainstay in the treatment of hematologic malignancies, offering remarkable efficacy and durable responses in various disease settings, often with manageable toxicity profiles (1, 2). However, CAR T-cell therapy is associated with a distinct spectrum of particular toxicities driven by immune system hyperactivation, triggered by downstream effects of CAR T-cell-target interactions, monocyte and macrophage stimulation, endothelial dysfunction, and release of proinflammatory cytokines. The associated toxicities include cytokine release syndrome (CRS), immune effector cell-associated neurotoxicity syndrome (ICANS), and immune effector cell associated hemophagocytic lymphohistiocytosis-like syndrome (IEC-HS) (3–5).

Despite advances in preventive and therapeutic strategies to reduce the risk of immune-related complications, such as the use of bridging therapies to shrink tumor burden before lymphodepletion, and early intervention upon symptom onset, non-relapse mortality (NRM) remains a concern. A recent meta-analysis reported unexpectedly high NRM rates, which vary depending on the specific CAR T-cell construct used (6).

Endothelial dysfunction is increasingly recognized as a key factor in the development and persistence of early CAR T cell–related toxicities. While the importance of endothelial health in immune-mediated complications is well established in allogeneic hematopoietic stem cell transplantation (allo-HSCT), its contribution to CAR T cell–associated toxicities is only beginning to be elucidated. Early HCT-related complications—such as sinusoidal obstruction syndrome (SOS), engraftment syndrome (ES), capillary leak syndrome (CLS), transplant-associated thrombotic microangiopathy (TA-TMA), acute graft-versus-host disease (aGVHD), and vascular idiopathic pneumonia syndrome (vascular-IPS)— all feature endothelial injury as a central pathophysiological component (7). Given the therapeutic similarities between HCT and CAR T cell therapy, together with the overlapping clinical manifestations of their toxicities, endothelial dysfunction emerges as a plausible shared pathogenic substrate in both settings.

This review aims to examine the role of endothelial dysfunction in CAR T-cell toxicities. We will first provide an overview of CAR T-cell structure, clinical use, and outcomes; then analyze the underlying pathophysiological mechanisms associated with endothelial injury, evaluate emerging biomarkers and scoring systems; and discuss potential therapeutic strategies targeting the endothelium-immune cell axis.

## 2 CAR T-cell structure, clinical use and outcomes

CAR T-cells are autologous T lymphocytes that are genetically engineered to express a chimeric antigen receptor targeting tumor-specific antigens. While new designs are being explored, second-generation CARs are still the most used. These usually include a single-chain variable fragment (scFv) derived from an antibody recognizing a particular antigen, linked to intracellular signaling domains such as CD3ζ and one costimulatory molecule (CD28 or 4-1BB), connected through a hinge and transmembrane region (1). Third-generation CARs combine both CD28 and 4-1BB domains to improve T-cell activation. In contrast, fourth-generation CARs, also called “TRUCKS” or “armored CARs,” add extra features like cytokine secretion and immune checkpoint regulation to enhance antitumor effects (3).

The clinical workflow for CAR T-cell therapy generally includes: 1) eligibility screening to confirm adequate organ function and performance status; 2) interruption or modification of disease-directed therapy to control tumor burden before apheresis (if necessary); 3) apheresis to collect mononuclear cells, which are sent for CAR T-cell manufacturing; 4) bridging therapy, when indicated, to prevent disease progression during the manufacturing period; 5) lymphodepleting chemotherapy, typically fludarabine plus cyclophosphamide administered over three consecutive days, to promote CAR T-cell expansion; and 6) CAR T-cell infusion (8, 9). Lymphodepletion is critical for *in vivo* CAR T expansion (10–12), and both the use of bridging therapy and achieving disease control before CAR T-cell infusion have been associated with improved outcomes (8, 12–14).

Currently, seven CAR T-cell products have received FDA approval. Two are directed against B-cell maturation antigen (BCMA) (idecabtagene vicleucel and ciltacabtagene autoleucel), for the treatment of multiple myeloma. Five target CD19: tisagenlecleucel, axicabtagene ciloleucel, brexucabtagene autoleucel, lisocabtagene maraleucel, and obecabtagene autoleucel. Additionally, numerous academic CAR T-cell products are in use in specific countries or centers (e.g., varnimcabtagene autoleucel and cesnicabtagene autoleucel in Spain (15), relmacabtagene autoleucel, equecabtagene autoleucel, and inaticabtagene autoleucel in China (16). CD19-directed CAR T-cell therapies are approved for a variety of B-cell malignancies, including B-cell acute lymphoblastic leukemia (B-ALL: tisa-cel, brexu-cel, obe-cel), diffuse large B-cell lymphoma (DLBCL: tisa-cel, axi-cel, liso-cel), mantle cell lymphoma (MCL: brexu-cel, liso-cel), follicular lymphoma (FL: tisa-cel, axi-cel, liso-cel), and chronic lymphocytic leukemia (CLL: liso-cel only).

CAR T-cell kinetics, clinical outcomes, B-cell aplasia duration, and persistence vary significantly across indications and products (2). In CD19-directed CAR T-cell therapy, B-cell aplasia is strongly correlated with CAR T-cell persistence (17). Generally, CD28-based constructs exhibit rapid expansion and shorter persistence, while 4-1BB-based products display slower expansion but longer *in vivo* survival. Other factors influencing persistence include T-cell subset composition (e.g., naive vs. memory T-cells), disease type, and the characteristics of the tumor microenvironment (TME) (2). Clinical outcomes vary across diseases. Reported overall response rates (ORR), complete response (CR) rates, median progression-free survival (mPFS), and overall survival (mOS) are summarized in **Table 1**.

**Table 1:** CAR T-cell clinical outcomes according to different diseases.

Disease	Overall response rates (ORR)	Complete response (CR) rates	Median progression-free survival (mPFS)	Overall survival (mOS)
B-ALL (17)	85-90%	85-90%	12 months	20 months
DLBCL (18–21)	80%	60%	6–15 months	15–25 months
MCL (22)	90%	70%	26 months	46 months
FL (23)	92%	74%	57 months	5 years
CLL (24)	80%	50%	18 months	43 months
MM (25)	85%	73%	*	*

B-ALL, B-cell acute lymphoblastic leukemia; DLBCL, diffuse large B-cell lymphoma; MCL, mantle cell lymphoma; FL, follicular lymphoma; CLL, chronic lymphocytic leukemia; MM, multiple myeloma.

*Not reached yet.

Although CAR T-cells are primary used for the treatment of malignancies, autoimmune diseases and even infectious disorders can also be targeted as well, with promising results emerging for autoimmune indications and several ongoing trials ongoing, mostly involving CD19-directed products (26, 27).

Several factors are associated with long-term responses to CAR T-cell therapy. Although these also vary by disease, lower tumor burden at the time of infusion is consistently associated with deeper responses and prolonged survival (2). Conversely, the presence of dense TME impairs CAR T-cell function and is linked to poorer outcomes (28–31).

## 3 CAR T-cell-associated toxicities

Cytokine release syndrome (CRS), immune effector cell-associated neurotoxicity syndrome (ICANS), and immune effector cell-associated hemophagocytic lymphohistiocytosis-like syndrome (IEC-HS) are well-recognized immune-mediated toxicities following CAR T cell infusion. These syndromes share a common initial pathophysiology, involving immune system hyperactivation, driven by the interplay between infused CAR T-cells and host immune cells, leading to a surge in proinflammatory cytokines and, in some cases, an associated coagulopathy. Other immune-related toxicities, such as immune effector cell-associated hematotoxicity (ICAHT), tumor inflammation-associated neurotoxicity (TIAN), and movement and neurocognitive treatment-emergent adverse events (MNTs), have also been described but fall outside the scope of this review (32–34).

### 3.1 Cytokine release syndrome

**CRS** is a systemic inflammatory syndrome characterized by elevated levels of circulating inflammatory cytokines, such as interleukin-6 (IL-6), interferon γ (IFN-γ) and IL-1, alongside widespread immune cell activation. CRS can occur in multiple clinical contexts, including infections, autoimmune conditions, malignancies, and other immunotherapies (35). It was first described following CAR T-cell therapy in 2010, in a patient with metastatic colon cancer treated with an ERBB2-targeted CAR T-cell product. After infusion, the patient developed respiratory distress, bilateral pulmonary infiltrates, and multiorgan failure, ultimately dying five days after despite intensive medical intervention. Serum cytokine analysis revealed high levels of IFNγ, granulocyte macrophage-colony stimulating factor (GM-CSF), tumor necrosis factor-α (TNF-α), IL-6, and IL-10 (36).

CAR T-cells become activated upon recognition of tumor-associated antigens, releasing large quantities of perforin, granzymes, and proinflammatory cytokines that induce pyroptosis (inflammatory cell death) in tumor cells (37–39). This process triggers the release of damage-associated molecular patterns (DAMPs), which activate monocytes and macrophages via pattern recognition receptors (PRRs), further amplifying the inflammatory response. Interleukin-1β (IL-1β), generated through inflammasome activation during macrophage pyroptosis, plays a critical role in sustaining and amplifying this cytokine cascade (38, 40–42).

Among the released cytokines, interleukin-6 (IL-6) is central to the systemic manifestations of CRS. IL-6, in combination with its soluble receptor, activates endothelial cells via the GP130 signaling pathway, increasing capillary permeability and driving the development of capillary leak syndrome, hypotension and coagulopathy (40). IL-6 receptor (IL-6R) blockade with tocilizumab -a monoclonal antibody targeting IL-6R- is a cornerstone of CRS management (43).

CRS is the most common early toxicity following CAR-T cell infusion (44). It usually occurs within the first 14 days post-infusion, with a median onset of 2–7 days. Fever is typically the first manifestation, followed by chills, sinus tachycardia, hypotension, hypoxemia, and dyspnea (32). Reported incidence ranges from 42% to 92%, with severe cases occurring in 0-24% depending on the CAR T-cell product (45–48). CRS-related mortality has been linked to respiratory failure, cardiac arrest, or multiorgan dysfunction (6).

Risk factors for CRS include both patient- and product-related variables. Patient-related risks encompass disease type, high tumor burden, active infection, low platelet count, elevated systemic inflammatory markers, and biomarkers of endothelial activation such as angiopoietin-2 and von Willebrand factor. Product-related risks include the use of CD28 costimulatory domains, high CAR T-cell dose or expansion kinetics, and fludarabine-based lymphodepletion regimens (3, 49).

CRS should be graded according to the 2019 American Society for Transplantation and Cellular Therapy (ASTCT) consensus criteria, based on fever, blood pressure, and respiratory status (4).

**Figure 1:**
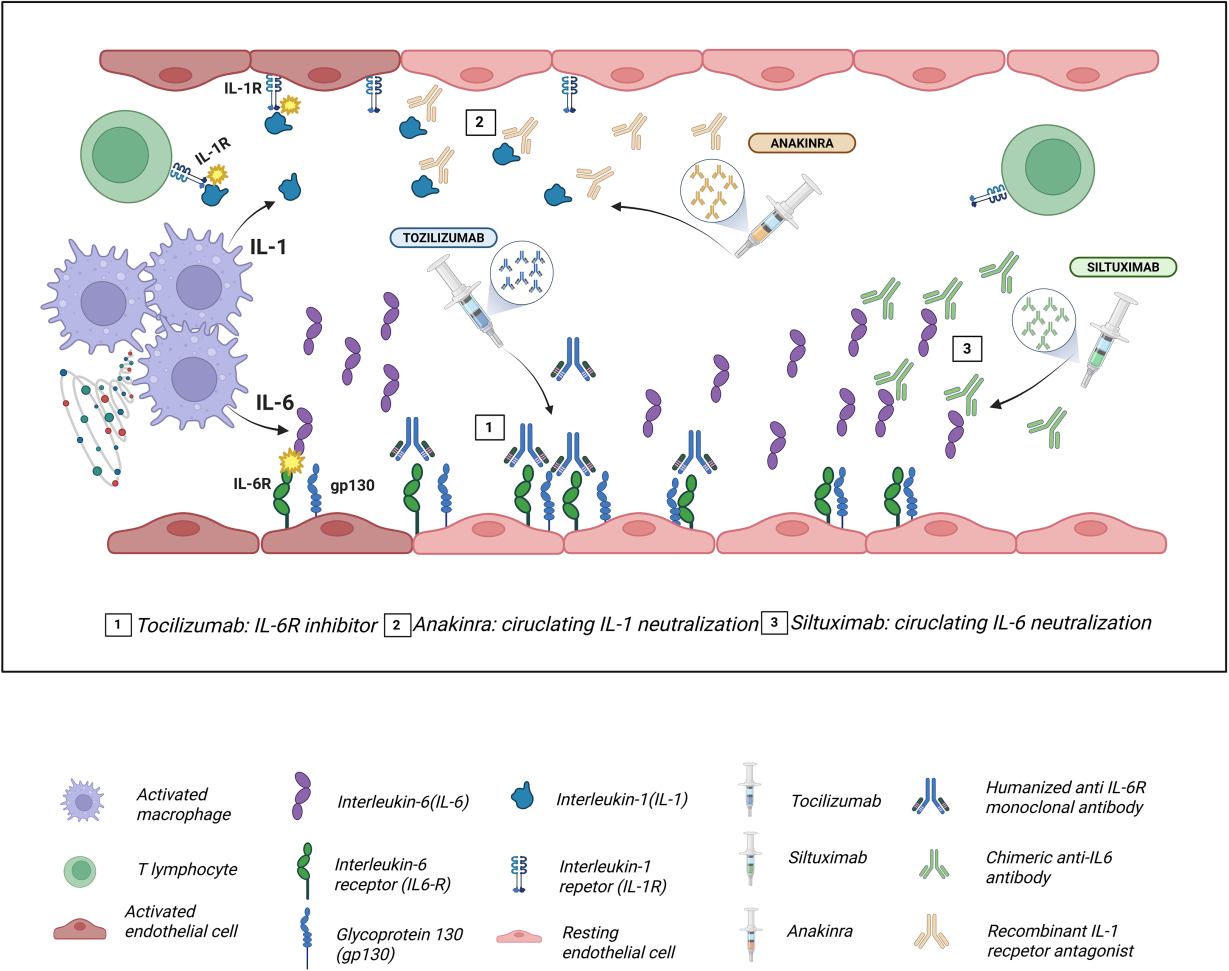
Molecular targets of the main drugs used in the management of cytokine release syndrome (CRS). Tocilizumab, a humanized monoclonal antibody against IL-6R, blocks both classical and trans interleukin-6 (IL-6) signaling, preventing Glycoprotein (gp130)/JAK-STAT3 activation and attenuating endothelial dysfunction, capillary leak, and hypotension characteristic of CRS. Siltuximab binds directly to circulating IL-6, neutralizing it and preventing its interaction with interleukin-6 receptor (IL-6R), thereby reducing systemic inflammatory amplification. Anakinra, a recombinant interleukin-1 receptor antagonist (IL-1R1a), inhibits IL-1α/IL-1β signaling via MyD88 and NF-κB, decreasing macrophage-mediated inflammation and contributing to the control of refractory CRS.

First-line treatment consists of supportive care and administration of tocilizumab, while corticosteroids are typically reserved for cases that are refractory to initial therapy. In severe or steroid-resistant presentations, additional agents such as anakinra (IL-1 receptor antagonist), siltuximab (anti-IL-6), ruxolitinib (JAK2 inhibitor), emapalumab (anti-INF-γ), antithymocyte globuline, and/or cyclophosphamide may be considered (50–54) (**Figure 1**).

### 3.2 Immune effector cell-associated neurotoxicity syndrome

**ICANS** comprises a spectrum of neurological manifestations, typically manifesting as encephalopathy characterized by inattention, disorientation, expressive aphasia, tremor, impaired fine motor skills, and decreased level of consciousness. Additional findings may include focal or generalized weakness, myoclonus, seizures, and, in severe cases, cerebral edema leading to increased intracranial pressure and brain herniation (40, 55).

While systemic inflammation contributes to ICANS, its pathophysiology is largely driven by blood brain barrier (BBB) disruption, increased vascular permeability, and glial cell injury (3). The BBB is a highly selective interface composed of specialized cerebrovascular endothelial cells, pericytes, and astrocytes that maintain central nervous system (CNS) homeostasis (56). Elevated circulating cytokines, more prominently IL-1, IL-6, TNF-α, interact with their specific receptors and activate proinflammatory signaling pathways such as NF-κB, MAPK and JAK-STAT (57, 58). This activation within the vascular endothelium triggers the release of Weibel-Palade bodies containing von Willebrand factor (VWF) and angiopoietin 2 (Ang-2) (59, 60). VWF promotes microthrombus formation and cerebral thrombotic microangiopathy (61), while Ang-2 inhibits the Tie2 signaling pathway, thereby destabilizing the endothelium and increasing BBB permeability, which facilitates cytokine entry into the CNS (62, 63).

Pericytes, which are essential for maintaining microvascular integrity, become stressed by cytokines and secrete IL-6 and VEGF-A (both permeability-inducing mediators), thereby amplifying endothelial injury (64). Moreover, Parker et al. identified CD19 and its chaperone CD81 in human brain pericytes, with sustained CD19 expression from development though adulthood. This variable expression may lead to off-target interactions between anti-CD19 CAR T-cells and pericytes, contributing to an enhanced BBB breakdown. Interestingly, the same study demonstrated that CD19 expression in mouse brain cells is markedly lower than in human brain cells, which may help explain the lower incidence of ICANS and highlights the translational limitations of these models (65).

Monocyte infiltration into the CNS and activation of resident microglia lead to the release of IL-1β, IL-6, and other pro-inflammatory mediators within the brain parenchyma (43). Astrocyte activation upon exposure to inflammatory cytokines further contributes to the development of cerebral edema, and IL-1β-induced VEGF-A disrupts endothelial tight junctions, exacerbating BBB leakiness (56). The accumulation of leukocytes and cytokines in the CNS promotes neuroinflammation, and *in situ* activation of microglia and macrophages induces the release of neurotoxic metabolites, such as glutamate and quinolinic acid, leading to neuronal excitotoxicity via NMDA receptor stimulation (40, 66).

ICANS incidence varies between 0-63%, with severe cases in up to 31% of patients (47, 48, 67–69), widely depending on the CAR T-cell product and disease context. Risk factors mirror those for CRS and include early-onset and severe CRS (70).

ICANS usually develops within the first 10 days after infusion, most often after CRS, though concurrent or independent onset has been reported (4, 58), as well as delayed presentations (71).

ICANS grading follows the ASTCT consensus, which includes four domains: the 10-point immune effector cell-associated encephalopathy (ICE) score (which should be routinely monitored after infusion), level of consciousness, presence and type of seizures, motor deficits, and signs of cerebral edema (4).

First-line treatment consists of supportive care and corticosteroids. In refractory cases, anakinra, siltuximab, and intrathecal or systemic chemotherapy may be considered (50–52, 54, 72, 73).

### 3.3 Immune effector cell-associated hemophagocytic lymphohistiocytosis-like syndrome

IEC-HS is a rare, life-threatening hyperinflammatory syndrome, phenotypically resembling secondary hemophagocytic lymphohistiocytosis (HLH) or macrophage activation syndrome, and is directly related to immune effector cell therapy. It is characterized by uncontrolled immune activation and multi-organ failure.

Pathophysiologically, IEC-HS results from sustained CAR T-cell and CD8+ T-cell activity, compounded by profound and persistent NK cell lymphopenia, which impairs immune regulation (74). This leads to high systemic levels of IL-1β, IFN-γ, and IL-18, in addition to IL-6, perpetuating pathological immune activation, including massive macrophage stimulation reflected in extreme hyperferritinemia, cytopenias, and multi-organ dysfunction.

CAR T-cell expansion in this setting may occur independently of antigen presence and it is associated with uncontrolled T cell proliferation. The distinct cytokine profile, combined with persistent NK lymphopenia, suggests IEC-HS represents a late-stage variant of CRS with a different immunopathogenesis. requiring therapeutic strategies beyond IL-6 blockade (74, 75).

Clinically, IEC-HS shares several features with CRS, such as fever and multiorgan dysfunction. However, it is particularly characterized by progressive or new-onset cytopenias, marked hyperferritinemia, coagulopathy (including hypofibrinogenemia) and/or elevated liver enzymes (74). IEC-HS often emerges during or after the resolution of CRS and should not be misclassified as severe CRS alone. The absence of standardized diagnostic criteria prior to recent consensus definitions likely contributed to its underrecognition (5). Efforts have been made to identify biomarkers capable of distinguishing IEC-HS from CRS (76); however, as these markers fall outside the scope of this review and are not primarily related to endothelial injury, they are not discussed in detail herein.

The syndrome also shares risk factors with CRS, including high tumor burden and elevated CAR T-cell doses, and is more frequently reported with CD22- or BCMA-targeted products than with CD19-directed constructs (74, 77).

Treatment includes corticosteroids and anakinra as first-line therapy. In refractory cases, escalation may include the addition of agents such as ruxolitinib, emapalumab, or low-dose etoposide (5).

### 3.4 Coagulopathy

Coagulopathy may develop in the setting of CRS, ICANS, or IEC-HS, as a result of widespread immune activation and endothelial injury. This culminates in a prothrombotic state characterized by Weibel–Palade body exocytosis, release of VWF and factor VIII, and exposure of tissue factor, initiating the coagulation cascade (78, 79). Enhanced thrombin generation leads to consumption of fibrinogen, clotting factors, and platelets, while secondary fibrinolysis elevates D‐dimer levels (63). Simultaneously, depletion of natural anticoagulants such as antithrombin and proteins C and S disrupts regulatory mechanisms, perpetuating microthrombosis and endothelial damage, exacerbating capillary leak syndrome and contributing to organ dysfunction (80, 81). In severe cases, the coagulopathy may fulfill diagnostic criteria for disseminated intravascular coagulation (DIC) (82).

Although endothelial dysfunction and coagulopathy are closely linked, overt consumption coagulopathy with clinical bleeding or thrombosis usually occurs only in patients with severe CRS or ICANS (56, 83). The highest risk of clinical coagulopathy is within the first month after infusion (84) and, while not frequent, its mortality rates are concerning (85).

## 4 The endothelium in CAR T-cell toxicities

Endothelial dysfunction has been identified as a cornerstone in the development and perpetuation of CAR T-cell-associated toxicities.

CAR T-cell recipients often show signs of endothelial dysfunction even prior to infusion. Most patients have undergone multiple lines of therapy, including cytotoxic agents known to cause endothelial injury. Additionally, a substantial proportion may have received prior allogeneic or autologous stem cell transplantation (SCT), which has been independently associated with endothelial damage (86, 87).

**Figure 2:**
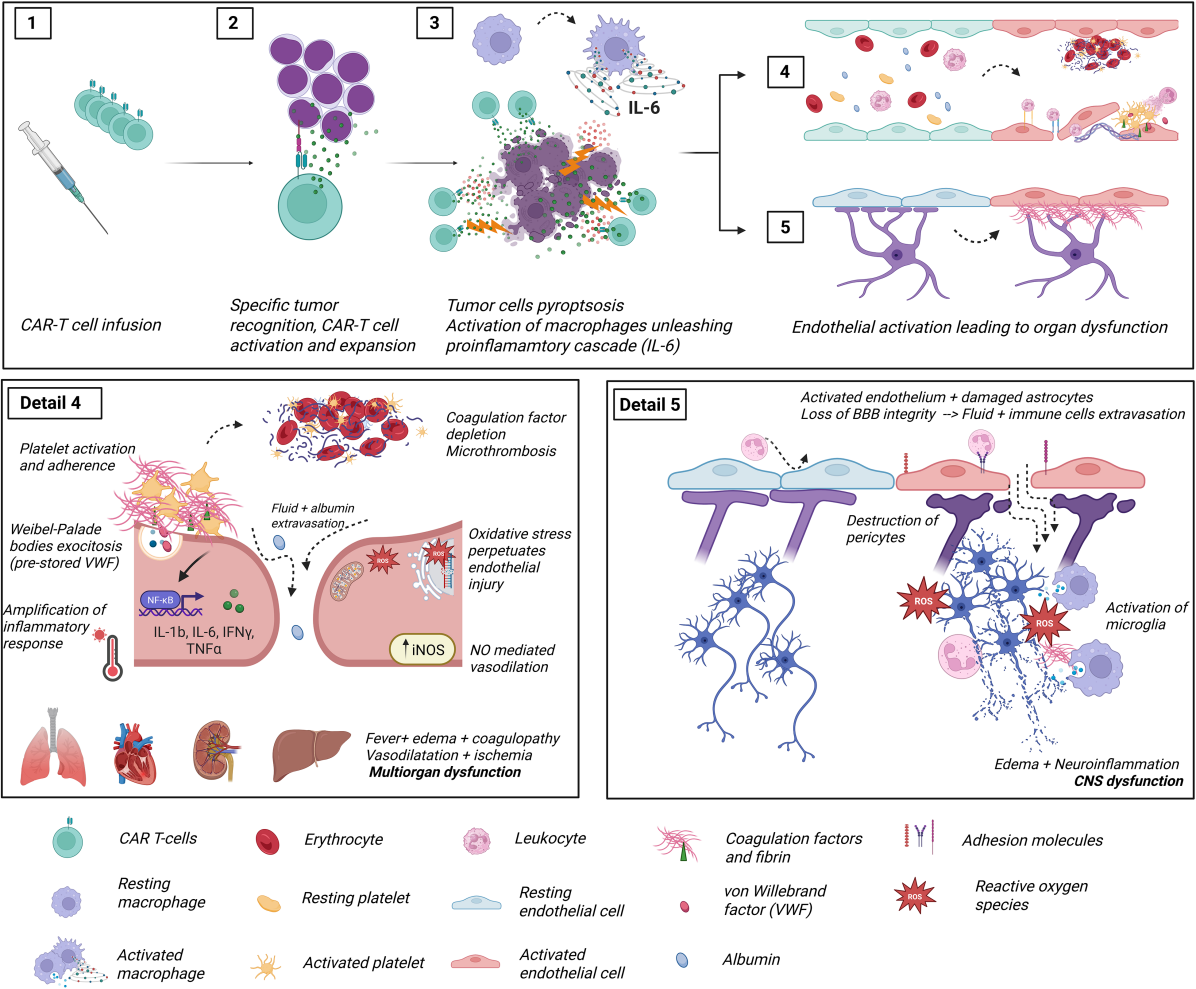
Diagram representing the pathophysiology of CAR-T cell treatment toxicities and its relationship with endothelial cells. Detail 1: CAR T-cells are infused into the patient. Detail 2: CAR T-cells recognize tumor antigens through their chimeric receptor, leading to activation and clonal expansion of the CAR T-cell population. Detail 3: Activated CAR T-cells release induce pyroptosis (inflammatory cell death) of tumor cells, triggering the release of high levels of damage-associated molecular patterns (DAMPs), which are recognized by pattern recognition receptors (PRRs) on host macrophages and monocytes to secrete additional cytokines (IL-6, TNFα…). These pro-inflammatory cytokines have an activating effect on ubiquitous endothelial layers. Detail 4: Longitudinal section of a blood vessel showing the normal disposition of an endothelial layer (blue cells). The red cells depict activated endothelial cells and their consequences (Panel 4 detail): endothelial cells lose their tight junctions, and fluid extravasation occurs. Additionally, dysfunctional endothelial cells switch their secretory phenotype of anticoagulant and fibrinolytic factors towards a hypercoagulative and hypofibrinolytic state due to increased release of PAI-1 and preformed TF and VWF. Intravascular microthrombi and liberation of neutrophil extracellular traps result in turbulent blood flow and agglutination of circulating cells, ultimately leading to tissue hypoperfusion. In addition, detached endothelial cells pass to circulation as Circulating Endothelial Cells (CECs), exposing the extracellular matrix, highly reactive to circulating platelets. The activation of iNOS increases NO availability, with both a vasodilatory effect in smooth muscle and oxidative cell damage. Ultimately, dysfunctional endothelial cells further amplify the cytokine release syndrome via upregulation of nuclear factor kappa B, which is linked to the inflammatory and oxidative stress responses. Detail 5: Longitudinal section of blood-brain barrier (BBB) showing analogous activation of endothelial cells responsible for ICANS. The pericytes, a mainstay of the BBB regulation, become inured following endothelial activation, leading to fluid and cell leakage into the cerebral parenchyma. This penetration of the central nervous system is followed by activation of microglia and neuroinflammation, responsible for the typical clinical picture of ICANS. This figure was created with BioRender.com (Diaz-Ricart, M. (2025) https://BioRender.com). CECs: circulant endothelial cells; CRS, Cytokine release syndrome; ICANS, Immune effector cell associated neurotoxicity syndrome; IFNγ, interferon gamma; IL, interleukin; iNOS, inducible nitric oxide synthase; NO, nitric oxide; NETs, neutrophil extracellular traps; PAI-1, plasminogen activator inhibitor 1; ROS, reactive oxygen species; TF, tissue factor; TNFα, tumor necrosis factor α; VWF, Von Willebrand factor.

Just before CAR T-cell infusion, lymphodepleting chemotherapy, typically a combination of fludarabine and cyclophosphamide, has also been associated to further endothelial damage (87–89). Finally, the cytokine storm that ensues after infusion exerts a direct and severe insult on the vascular endothelium (90). As described previously in the pathophysiology of CRS, ICANS, and IEC-HS, endothelial activation and damage play a central role in mediating clinical toxicity. The interconnected pathways linking endothelial dysfunction with CRS and ICANS are illustrated in **Figure 2**.

Endothelial cells exposed to inflammatory stimuli undergo a phenotypic shift. They upregulate adhesion molecules for leukocytes and platelets, such as vascular cell adhesion molecule-1 (VCAM-1) and VWF, respectively, while also increasing the expression of pro-angiogenic mediators, such as vascular endothelial growth factor (VEGF) and angiopoetin-2 (Ang-2). Simultaneously, the intercellular junction protein VE-Cadherin is downregulated, and the synthesis of key vasodilators and antiplatelet agents, such as prostacyclin and nitric oxide, is impaired. These molecular changes result in an endothelium that is procoagulant, proinflammatory, hyperpermeable, vasoplegic, and prone to excessive proliferation (91, 92).

The clinical manifestations of endothelial dysfunction vary depending on the severity of the insult and the anatomical site of endothelial injury (87).

There are mechanistic similarities between the endothelial damage observed in CAR T-related toxicities and that seen after allo-SCT, including the release of common inflammatory cytokines (e.g., IL-1, IL-6, TNFα), followed by enhanced leukocyte adhesion and migration due to increased expression of endothelial adhesion molecules (93).

## 5 Circulating biomarkers and surrogate indices of endothelial dysfunction, hemostatic imbalance, and innate immune activation in CAR T-cell toxicities

Multiple biomarkers reflecting endothelial dysfunction and associated pathways, including coagulation and complement activation, have been studied to elucidate their role in the pathogenesis of CAR T-cell toxicities and to assess their potential predictive value for toxicity severity and treatment outcomes. In this section, we review key laboratory parameters and classify them into the following categories: (1) biomarkers of endothelial dysfunction, (2) surrogate indices of endothelial stress, (3) biomarkers of coagulopathy (basic coagulation parameters and activators, fibrinolysis parameters and fibrin degradation products, and primary hemostasis parameters), and (4) markers of innate immunity activation. This framework aims to facilitate understanding of the molecular networks that contribute to CAR T-cell–related complications and their potential clinical utility.

### 5.1 Biomarkers of endothelial dysfunction

#### 5.1.1 Angiopoietin-Tie2 axis and angiogenic factors

The angiopoietin axis, comprising angiopoietin-1 (Ang-1), angiopoietin-2 (Ang-2), and their ratio (Ang-2:Ang-1) has been implicated in the development of CAR T-cell toxicities. Both ligands bind to the Tie2 receptor tyrosine kinase on endothelial cells, with opposing effects: Ang-1 promotes endothelial stability, while Ang-2, released in response to endothelial injury, destabilizes cell-cell junctions, enhances vascular permeability, and induces proliferative signaling (94). Elevated Ang-2 levels have been associated with severe CRS and were already detectable at the time of lymphodepletion in patients who subsequently developed severe toxicity (49). Increased post-infusion Ang-2 levels also predicted the need for ICU admission (95), and the Ang-2:Ang-1 ratio correlated with organ dysfunction in severe CRS cases (96). Growth differentiation factor-15 (GDF-15) is a stress-responsive cytokine produced by cardiomyocytes, endothelial cells, and other cell types in response to metabolic or oxidative stress, inflammation or cellular senescence (97). GDF-15 has been found elevated in CAR T-cell recipients compared to healthy controls and in this setting is considered a marker of endothelial injury (98).

#### 5.1.2 TNF receptor I

TNFRI, a receptor for TNF-α, is upregulated in endothelial cells in response to inflammation. Its engagement leads to NF-κB and AP-1 (two families of transcription factors) activation, resulting in increased expression of leukocyte adhesion molecules (99). Although TNFRI is not endothelial- specific, elevated circulating levels have been observed at the onset of CAR T-cell toxicities compared to post-infusion values (95).

#### 5.1.3 Adhesion molecules and endothelial junction proteins

Activated endothelial cells upregulate proteins involved in leukocyte adhesion and transmigration, including VCAM-1 and intercellular adhesion molecule-1 (ICAM-1). Soluble forms of these molecules have been found elevated in patients with severe CRS (96) and coagulopathy (81, 100). In contrast, platelet endothelial cell adhesion molecule (PECAM-1), which maintains intercellular junction integrity, is typically downregulated during endothelial stress, contributing to increased vascular permeability.

#### 5.1.4 Von Willebrand Factor

VWF is a multimeric protein with multiple functions, most prominently mediating platelet adhesion to the exposed subendothelium, promoting platelet-platelet interactions, and stabilizing coagulation factor VIII. It is released from Weibel-Palade bodies in response to endothelial injury (101). Several studies have demonstrated that VWF levels increase during CRS and correlate with its severity (80, 96). Notably, elevated VWF levels at the time of lymphodepletion have been associated with subsequent CRS development (49). More recently, VWF has been proposed as part of a biomarker panel to differentiate CRS from sepsis, another common and serious early complication following CAR T-cell infusion (95).

#### 5.1.5 Soluble Suppression of tumorgenicity 2 protein

ST2 is a member of the interleukin-1 (IL-1) transmembrane receptor superfamily and is expressed by various cell types, including endothelial cells (102). Its soluble form (sST2) acts as a decoy receptor for IL-33, impairing its anti-inflammatory and antifibrotic signaling. sST2 is a recognized marker of cardiovascular stress and inflammation (103). In CAR T-cell patients, sST2 levels increased significantly at the onset of toxicities and showed predictive potential for treatment-related complications (95).

### 5.2 Surrogate indices of endothelial dysfunction: EASIX and related scores

Direct measurement of endothelial biomarkers is not routinely available in clinical practice, as these assays may be time-consuming or require specialized platforms. Therefore, surrogate indices derived from standard laboratory parameters have been proposed to approximate endothelial dysfunction in the context of CAR T- cell therapy.

#### 5.2.1 EASIX

The Endothelial Activation Stress Index (EASIX) is calculated using the formula: (LDH [U/L] x Creatinine [mg/dL])/Platelet count [10^9^/L]. Although composed of nonspecific variables that may be altered by multiple causes in complex hematologic patients, EASIX has emerged as a valuable surrogate marker of endotheliopathy. Originally developed in the setting of allo-HSCT, EASIX has consistently shown predictive value for complications and poor outcomes (104–106). More recently, its application has been extended to CAR T-cell therapy. Several studies have demonstrated that elevated pre-infusion EASIX scores correlate with the development and severity of CAR T-cell-related toxicities (107–109).

#### 5.2.2 Modified and simplified EASIX (m-EASIX, s-EASIX)

In an effort to improve performance or simplify calculation, various adaptations of the EASIX formula have been evaluated. The modified EASIX (m-EASIX) replaces creatinine with C-reactive protein. It has shown good predictive accuracy for severe CRS, ICANS (110–112) and consumptive coagulopathy (80). The simplified EASIX (s-EASIX) omits creatinine from the formula. Despite its simplicity, it has demonstrated utility in predicting severe CRS (94, 107, 113) and ICANS (114), although its validation has often been limited to specific patient subsets and CAR T-cell constructs (107, 115).

#### 5.2.3 EASIX-based cytokine augmented models

Building upon the original index, some authors have incorporated inflammatory biomarkers, such as IL-6, IL-10, or ferritin, into EASIX-based models. These scores considering cytokine levels have also demonstrated predictive value for early identification of patients at risk for severe CRS, ICANS, and coagulopathy (116–118).

### 5.3 Biomarkers of coagulopathy

These biomarkers can be grouped into three main categories: basic coagulation parameters and activators, fibrinolysis-related markers and fibrin degradation products, and markers of primary hemostasis.

#### 5.3.1 Basic coagulation parameters and activators

Prolonged prothrombin time (PT) and activated partial thromboplastin time (aPTT) have been observed in patients with CRS, correlating with its severity (80, 83, 100). These alterations reflect the consumption of coagulation factors during systemic inflammation. Additionally, fibrinogen levels may decrease significantly in patients with severe CRS (83, 84) or IEC-HS (119, 120), where hypofibrinogenemia constitutes a diagnostic criterion (121). Of note, low fibrinogen concentrations have also been reported following tocilizumab administration (122).

Other studies have demonstrated that patients experiencing severe CRS or ICANS exhibit decreased levels of antithrombin (80), elevated tissular factor expression (81), and increased thrombin generation, as reflected by higher plasma levels of prothrombin fragment 1 + 2 (100). These findings confirm the presence of dysregulated coagulation and its mechanistic link to endotheliopathy in promoting a prothrombotic state.

#### 5.3.2 Fibrinolysis markers and fibrin degradation products

Activation of the coagulation cascade triggers a compensatory response by the fibrinolytic system, where plasmin degrades fibrin clots. The balance of fibrinolytic activity is critical in inflammatory conditions such as CRS and IEC-HS.

The soluble urokinase-type plasminogen activator receptor (suPAR) is the soluble form of uPAR, a glycoprotein expressed in the membrane of immune cells. It is a marker of immune activation and fibrinolysis, generated upon the binding of urokinase plasminogen activator (uPA) to its membrane receptor (uPAR), leading to suPAR release into plasma. Active uPA cleaves plasminogen into plasmin but also activates the complement system (123). Although suPAR levels may vary across clinical settings (124, 125), in CAR T-cell recipients they have been shown to be elevated compared to controls, and to correlate with m-EASIX scores pre-infusion, and EASIX scores at two weeks post-infusion (98).

Plasminogen activator inhibitor-1 (PAI-1) is a serine protease inhibitor produced by endothelial cells and platelets. By inhibiting uPA and tissular plasminogen activator (t-PA), it exerts a strong antifibrinolytic effect. Elevated PAI-1 levels have been proposed as a potential biomarker to distinguish CRS from sepsis, being significantly higher in the latter (95).

As a result of increased fibrinolysis, fibrin degradation products (FDPs) such as D-dimer are commonly elevated in high-grade CRS (80, 83, 84, 100), reflecting the downstream effects of uncontrolled thrombin and fibrin generation.

#### 5.3.3 Primary hemostasis markers

The relationship between endothelial damage and activation of primary hemostasis is particularly evident following the enhanced release of VWF into the plasma and extracellular space. This promotes platelet adhesion and aggregation, ultimately leading to platelet consumption. Thrombocytopenia has been observed in patients with severe CRS, particularly in those with coexisting laboratory evidence of coagulopathy (80).

ADAMTS-13 (A Disintegrin and Metalloproteinase with thrombospondin type 1 motif, member 13) is an enzyme that cleaves ultra-large VWF multimers and regulates platelet adhesion. Lower levels of ADAMTS-13 have been observed in patients with severe ICANS compared to those with milder forms (56), as well as at post-infusion time compared to baseline values in CAR T- cell recipients. These findings suggest a role for microangiopathic features in the pathogenesis of neurotoxicity and other thrombotic complications.

### 5.4 Biomarkers of innate immune activation

CAR T-cell-associated toxicities involve not only endothelial dysfunction and coagulation disturbances, but also profound activation of the innate immune system. Inflammatory cytokines, complement activation and neutrophil-derived mediators all contribute to the immunopathogenesis of toxicities such as CRS, ICANS, and IEC-HS.

#### 5.4.1 Complement activation

The alternative complement pathway can be initiated by cytokine-induced neutrophil activation and subsequent C3b release (126), as well as by coagulation-derived proteases such as thrombin and plasmin, which can cleave complement components to generate the anaphylatoxins C3a and C5a. Terminal complement activation results in the formation of the membrane attack complex (MAC, C5b9). Its soluble form, sC5b9, has been shown to be elevated in CAR T-cell patients compared to healthy controls and correlates with both EASIX and m-EASIX scores at different time points (98). Moreover, biomarker panels including sC5b9 have demonstrated strong performance in predicting severe CAR T-cell-related toxicities and in differentiating CRS from sepsis (95). Recently, increased sC5b9 have also been associated with grade ≥ 2 ICANS and higher m-EASIX scores (127).

#### 5.4.2 Neutrophil extracellular traps

NETs are web-like structures composed of DNA, histones, and granule proteins released by neutrophils in response to inflammatory stimuli. Originally described as part of the antimicrobial response, NETs also play a pathological role in cancer and immunotherapy-related syndromes. In the tumor microenvironment, NETs promote immune suppression and tumor progression. In the context of CAR T-cell therapy, they may physically impede CAR-T function (128, 129).

NETs have been observed to increase after CAR T-cell infusion and have emerged as promising biomarkers for severe toxicity prediction. They have also shown utility in distinguishing CRS from sepsis, particularly when included as part of multiparameter biomarker panels (95).

## 6 Potential clinical applications of biomarkers

The potential clinical applications of the biomarkers discussed above are summarized in **Table 2**. Biomarkers have been categorized according to their underlying biological process (endothelial dysfunction, hemostatic imbalance, and innate immune activation including complement pathways), and their proposed clinical utility. These include prediction of mortality, prediction of toxicity onset and severity, and differential diagnosis, particularly to distinguish CAR T-toxicities from clinically overlapping syndromes such as sepsis).

**Table 2:** Circulating biomarkers and surrogate scores of endothelial dysfunction, hemostasis misbalance, and other linked pathways, and their potential clinical uses in each moment during CAR T-cell immunotherapy.

Immunotherapy time point	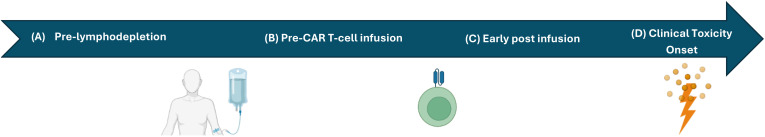			
Potential use → Type of biomarker ↓	Mortality prediction	Toxicity prediction	Differential diagnosis clinically overlapping syndromes	Toxicity severity stratification
Endotheliopathy	m-EASIX at A (80)	VWF, Ang-2:Ang-1 ratio* at A (49) Ang-2:Ang-1 ratio^&^ at A (56) sST-2*^&^ at C (95) High Ang-2:Ang1*, sVCAM-1* at C (96) m-EASIX* at A (130) EASIX*^&^, m-EASIX*^&^ at A, B and C (111, 112) EASIX*^&^, m-EASIX*^&^ and s-EASIX*^&^ at A (108) EASIX-F*^&^, EASIX-FC^*&^ at A (118) EASIX^&^ and m-EASIX^&^ at A and B (131) EASIX*^&^, m-EASIX*^&^, s-EASIX*^&^ at A, B and C (111) EASIX*^&^, EASIX-F*^&^, EASIX-FC*^&^, sEASIX*^&^ at C (114) m-EASIX^*#^ at A (80)	Ang-2*, VWF* at point D (95)	VWF*, Ang-2*, Ang-2:Ang-1 ratio* at D (51) sPECAM-1* at C (74) Low Ang-1* High VWF*, Ang-2*, Ang-2:Ang1*, sE-selectin*, sICAM-1*, sVCAM-1* at C (84) sST2*, Ang-2* (95) at C VWF* at C (73) Ang-2:Ang-1 ratio^&^, VWF^&^, loss of VWF HMWM^&^ at C (58) High Ang-2:Ang-1 ratio^&^ at C (66) Ang-2^#^, Ang-2:Ang-1 ratio^#^, VEGF^#^ and s-VCAM-1^#^ at C and D (100) EASIX*^&^, m-EASIX*^&^ at A, B and C (111, 112) EASIX*^&^ at B (108) EASIX-F*^&^, EASIX-FC^*&^ at A (118) EASIX*^&^, m-EASIX*^&^, s-EASIX*^&^ at A, B and C (111)
Hemostasis imbalance		PT^&^, aPTT^&^, D-dimer^&^ Fibrinogen^&^ at B (132)	PAI-1^*&^ at point D (95) Hypofibrinogenemia^$^ at C (5)	PT*, aPTT*, fibrinogen*, D- Dimer*, platelets* at C (49) D-Dimer*, FDP*, PT*, aPTT*, TF* and Low fibrinogen* at C (81) PT*, aPTT*, fibrinogen*, D-dimer*, FVIII*, decreased platelet count*, decreased AT* at C (80). PT^&^, aPTT^&^, D-dimer^&^, platelet count^&^, fibrinogen^&^ at C, and Low ADAMTS-13:VWF ratio^&^ at D (56) PT^#^, D-dimer^#^, TF^#^, prothrombin fragment F1 + 2^#^ at C and D (100) PT^#^, aPTT^#^, Fibrinogen^#^, FDP^#^, TT^#^ at C (83) aPTT*, PT*, Fibrinogen*, platelets* at C (82).
Innate immune response and complement activation			sC5b9*, NETs* at point D (95)	sC5b9*, NETs* at point D (95)

ADAMTS-13, a disintegrin and metalloproteinase with a thrombospondin motif repeats 13; Ang-1, angiopoietin 1; Ang2, angiopoietin 2; aTPP; activated partial thromboplastin time; AT, antithrombin; CRS, cytokine release syndrome; EASIX, Endothelial activation stress index, m-EASIX, modified EASIX; EASIX-F, EASIX with ferritin; EASIX-FC, EASIX with ferritin and C reactive Protein; FDP, fibrin degradation products; HMW, high molecular weight multimers; ICANS, immune-effector cell neurotoxicity syndrome; NETs, neutrophil extracellular traps; PAI-1, plasminogen activator inhibitor 1; sPECAM, soluble platelet endothelial cell adhesion molecule; PT, prothrombin time; sC5b9, soluble C5b9 complement complex; sICAM-1, soluble intercellular cell adhesion molecule 1; sST2, Soluble suppression of tumorigenesis-2; sVCAM-1, soluble vascular cell adhesion molecule 1; TF, tissular factor; TT, thrombin time; VEGF, vascular endothelial growing factor; VWF, von Willebrand Factor.

Useful for prediction of Cytokine Release Syndrome (CRS)*, Immune effector cell-associated Neurotoxicity Syndrome (ICANS)^&^, Clinical coagulopathy^#^, Immune effector cell-associated hemophagocytic lymphohistiocytosis-like syndrome (IEC-HS)^$^.

The time point when these biomarkers demonstrated to be useful during immunotherapy pathway is indicated with letters A, B, C, D referencing pre-lymphodepletion, Pre-CAR T-cell infusion, early post-CAR T-cell infusion and toxicity onset, respectively.

Moreover, the utility of these biomarkers has been mapped across distinct time points in the CAR T-cell therapy timeline: pre-lymphodepletion, between lymphodepletion and CAR T-cell infusion, early post-infusion period, and toxicity onset.

## 7 Future perspectives and conclusions

As observed in complications that appear early after other forms of cellular therapy (7), the endotheliopathy and its related pathways are critical in the pathophysiology of CAR T-cell-associated toxicities. The key points of the present review are as follows:

- There is compelling evidence supporting the utility of circulating biomarkers for diagnostic confirmation, early prediction, severity stratification, and even non-relapse mortality prognosis.
- Most of the biomarkers reviewed remain confined to research settings. Their translation into clinical practice will require rigorous analytical validation, inter-laboratory standardization, and the development of evidence-based clinical guidelines delineating their diagnostic and therapeutic relevance.
- Growing recognition of the endothelium as a therapeutic target has prompted the evaluation of pharmacological agents with endothelial-protective properties for the management of CAR T-cell toxicities (133).
- To date, most interventional strategies involving endothelial modulation have focused on enhancing vascular permeability to facilitate CAR T-cell infiltration into solid tumors (134–136).
- The therapeutic strategies, aimed at stabilizing the endothelium, may offer a promising avenue to mitigate immune-mediated toxicity. These strategies may involve modulation of: proinflammatory cytokines, reactive oxygen species (ROS), adhesion molecules, and complement components (137).
- None of the mentioned interventions has reached advanced clinical development. However, several are currently under evaluation in investigator-initiated trials (138, 139).
-
   o Defibrotide, despite exhibiting a favorable safety profile, showed limited efficacy in preventing ICANS in patients treated with axicabtagene ciloleucel (139).
   o Statins, when initiated before immunotherapy and maintained during treatment, and combined with intrathecal dexamethasone, were associated with a reduction in the incidence and severity of ICANS (140).
   o Complement inhibitors have shown potential for the treatment of immunotherapy-related thrombotic microangiopathy (141).
   o *In vitro*, the combined blockade of TNFα and IL1β using adalimumab and an anti-IL-1β antibody, respectively, demonstrated a synergistic effect in preventing endothelial activation, warranting further clinical investigation (138).
   o Fourth-generation CAR T-cell constructs may carry an increased risk of immune-related toxicities. However, certain designs aim to reduce these effects by incorporating negative-feedback loops: a tocilizumab-secreting CAR T-cell design was shown to lower toxicity while unexpectedly increasing efficacy (142). Another promising approach involves spatially confined CAR T-cell activation and cytokine release, which could be advantageous in selected tumor types or anatomical locations. In a recent study, anti-CD19 CAR T-cells engineered to secrete IL-12 specifically within hypoxic environments achieved high efficacy rates and no detectable toxicity in DLBCL mouse models (143). To date, however, none of these strategies have been clinically validated as safer alternatives to second-generation CAR T-cells.
- The incorporation of biomarkers into clinical decision-making, including patient selection, preemptive toxicity management, and monitoring of endothelial-targeted therapies, remains an unmet need and a promising area of future research and personalized immunotherapy.
- In parallel, integrating multi-omic approaches with artificial intelligence–based predictive models holds great potential to refine risk stratification and develop more robust, individualized predictors of CAR T-cell related toxicities (144).
